# The impact of intergenerational support on the mental health of older adults: a discussion of three dimensions of support

**DOI:** 10.3389/fpubh.2025.1467463

**Published:** 2025-04-17

**Authors:** Kang Ren, Jing Lan, Lingyu Ge, Lei Zhou

**Affiliations:** College of Public Administration, Nanjing Agricultural University, Nanjing, China

**Keywords:** intergenerational support, mental health, older adults, CHARLS, financial support, daily care, emotional comfort

## Abstract

**Introduction:**

This paper examines the effects of intergenerational support on the mental health of older adults and elucidates the mechanisms underlying these effects.

**Methods:**

Utilizing data from the 2020 China Health and Retirement Longitudinal Study (CHARLS), We examine the effects of different dimensions of intergenerational support on the positive and negative emotions of older adults based on the main effect model and the buffer model. Then we employ instrumental variable methods to address key endogeneity concerns. The indirect effect mechanism through which children’s economic support influences the mental health of the older adults was examined using a mediation model. In addition, robustness tests and heterogeneity analyses were conducted.

**Results:**

The intergenerational support from children has a significant impact on the mental health of older adults. All three types of support contribute to the positive emotions of older adults. In terms of the impact on negative emotions, the regression coefficients for financial support and emotional support are significantly negative, while the direction of the effect of caregiving support is positive. Additionally, financial support promotes the mental health of the older adults by influencing their social participation. Heterogeneity analysis further indicates that the impact of intergenerational support varies across different subpopulations, with rural older adults particularly benefiting from emotional support.

**Discussion:**

Our findings reveal that financial support from adult children enhances older adults’ mental health by improving positive emotions and reducing negative emotions, and emotional support from children has a similar effect. However, while caregiving by adult children significantly boosts positive emotions among older adults, long-term caregiving also engenders feelings of guilt, which in turn exacerbates negative emotions and diminishes overall mental health. Our study emphasizes the need to consider Filial Piety Culture, social care support and social participation in order to improve older adults’ mental health.

## 1 Introduction

In the past decade, the age structure of China’s population has changed, and its population development is showing a declining birthrate and an aging population. According to the 2020 census, the proportion of people aged 65 and above in China reached 13.5%, an increase of 4.63 percentage points compared to the previous census. As population aging intensifies, there is growing concern about the health issues of the older adults, particularly their mental health. Depression among older adults has become a growing concern in China, with the prevalence of high-risk depression among older adults reaching 43.5% in 2018 (1). There are significant differences in mental health levels among different groups of older adults.

The “Law on the Protection of the Rights and Interests of the Older adults” in China stipulates that adult children have the obligation to support their parents, formally establishing the responsibility of adult children to care for their parents. The long-standing cultural tradition of filial piety and the concept of raising adult children for old age further influence family relationships on a non-institutional level. Thus, family-based care remains the primary mode of older people care in China, with intergenerational support from adult children serving as an important guarantee for the well-being of the older people in their later years. Scholars classify intergenerational support from adult children as informal care. Informal care can have positive mental health consequences due to emotional gains from taking social responsibilities and caring for loved ones, which is covered by the “expansion hypothesis” (2). Intra-family intergenerational support has become a significant factor affecting the mental health of the older adults, prompting many scholars to conduct empirical research on this topic. Some studies have found that informal caregiving from adult children has a significant impact on satisfaction (3–5) or on depression (6, 7), while others have demonstrated that informal care can be both a burden and a source of satisfaction simultaneously. In summary, there is a lack of consensus because intergenerational caregiving may have both positive and negative effects, as well as mutually reinforcing or offsetting impacts on the mental health of older persons through various channels of influence (8).

Exploring how intergenerational support affects the mental health of the older adults requires considering the support forms. Economic support, daily care, and emotional support are the basic dimensions of support that adult children provide to their parents, and the mechanisms of their impact on the recipients vary. The main effect model and buffer model are often used to explain the effects of intergenerational support on the mental health of the older adults. Adult children’s support for their older adult parents can improve life satisfaction or positive emotions and cushion the damage of stressful events to health (3, 4, 9–12). The effect of intergenerational support on mental health should be the comprehensive result of main effect and buffer effect. However, the existing research lacks in-depth discussion on the mechanism of action of the three types of support, making it difficult to untangle the effect of intergenerational caregiving. We add two key innovations to the existing literature: The first innovation is to describe mental health indicators from two dimensions, negative emotion and positive emotion, to enrich the hierarchy of mental health of the older adults.

The impact of social support on mental health can be indirect. Intergenerational financial support may indirectly improve physical health by improving social relationships and increasing opportunities for older adults to participate in social activities (13). Does this indirect effect have any effect on mental health? The role of economic support in promoting social participation have been discussed (13, 14), and so has the role of social participation in mental health (14, 15). This paper attempts to discuss the possible mediating mechanism between these two relationships. The second innovation is to explore how economic support affects the mental health of the older adults through enhancing social interaction.

The majority of extant literature was carried out in higher-income countries, with limited evidence from China. However, China provides a valuable test case of the impact of intergenerational caregiving on old careers’ mental health. Owing to the traditional concept of raising adult children for old age, older parents have practical expectations of support from their adult children (16). Yet, such a function of informal care has been gradually weakened by the simplified family structure and the work pressures of adult children. On the other hand, the economic and social transformation has also impacted the “culture of filial piety,” and parents’ expectations for their adult children’s old-age support and the “sense of shame” that brings burdens to their adult children may coexist. The third innovation lies in uncovering the dual mechanism at play. It highlights how filial satisfaction fosters positive emotions, while burden-related guilt triggers negative emotions. Given the significant differences in economic development levels and social policies between urban and rural areas in China, as well as the variations in support needs arising from different health conditions, gender, and income levels, we discuss the possible effect heterogeneity in the impact of intergenerational support on the mental health of older individuals. The analysis focuses on distinctions based on urban or rural status, health conditions, gender, and family income levels.

## 2 Literature review

As members of society, individuals establish various social relationships, and different stages of life are dominated by corresponding social relationships that influence their mental health (17). For the older adults, after retirement, external social relationships such as those with colleagues and friends gradually weaken, while internal family relationships, particularly those with adult children, begin to dominate. Adhering to the strong cultural tradition of filial piety, an essential part of Confucianism, caregiving for older parents is considered the adult children’s responsibility, obligation, and also a virtue (2). Intergenerational support from adult children has a significant direct impact on the mental health of the older adults, with those receiving such support often exhibiting better mental health (18).

In recent years, studies on the impact of intergenerational support on the mental health of the older adults boom, which often measure intergenerational support based on three aspects: economic support, life care and emotional comfort (12, 19).

Thus, the health effects of caregiving may vary by the intensity of the role, competing demands, relationship with the person cared for, availability of supports, and individual vulnerabilities, including health status prior to becoming a caregiver (20, 21).

Adult children’s economic support primarily affects the mental health of the older adults by enhancing positive emotions and reducing negative emotions. Older adults who receive financial support have higher life satisfaction (9) or lower levels of depression (11), and intergenerational financial support from adult children improves life satisfaction in older adults (4, 10). Additionally, adult children’s financial support improves the quality of life for the older adults, indirectly fulfilling other needs and contributing to better mental health (3, 19). Intergenerational financial support from adult children can also reduce the incidence of depression among the older adults (11), by alleviating anxiety and depression.

The impact of adult children’s daily care on the mental health of the older adults is controversial, with no consensus in the academic community. One view is that Daily care from adult children can reduce depression levels and improve the quality of life for the older adults (19), significantly promoting mental health (22). Scholars using CHARLS data and data from Vietnam and Myanmar have reached the same conclusions (23, 24). Another view argues that daily care has a significant negative impact on the mental health of the older adults. For healthy older individuals, daily care from adult children may undermine their confidence (25), affect their attitude towards aging, and compromise their independence (26), negatively impacting their mental health.

Intergenerational support can bring good mental health to older adults, and emotional support can promote mental health in older people more than daily care and financial support (3). More frequent family visits are associated with lower rates of depression (5). Regular interactions with adult children fulfill the emotional needs of older adult parents, help them manage their emotions, reduce psychological stress, and alleviate the risk of loneliness and depression (14, 27–29). These will improve their mental health (30, 31), something that economic support alone cannot achieve (32).

Active aging suggests that participation in social activities is a core pillar for ensuring the mental health of the older adults. Older individuals who frequently participate in social activities have higher subjective well-being and quality of life (33), and negative emotions such as depression and loneliness can be alleviated through social participation (15, 34). Multiple empirical studies have found that participation in social activities can significantly enhance the mental health levels (15, 35) or subjective well-being (36) of older individuals. Furthermore, family support can also promote the participation of older adults in social activities (36–38). Impact of social support on the mental health or life satisfaction of older individuals often exhibit group heterogeneity. Previous literature discusses this heterogeneity from various dimensions, including country or region (36, 39, 40), gender (29, 39, 41), income (40), and whether living with adult children (36). Given the differences in social support mechanisms and economic development levels between urban and rural areas in China, empirical research often focuses more on urban–rural or household registration heterogeneity (7, 41, 42), indicating that the effects of three types of intergenerational support on the mental health of urban and rural older individuals differ.

To sum up, existing research has primarily explored the influence of intergenerational child support on the mental health of the older adults from three dimensions: financial support, care support and emotional comfort support. However, how does different types of intergenerational support affect the mental health of older adults has not been considered. Moreover, is the main effect of intergenerational support on mental health in the older adults consistent with the stress buffering effect? Does intergenerational support indirectly affect the mental health of older adults? Is there population heterogeneity in this effect? These unresolved questions drive this article, which categorizes the mental health of the older adults into positive and negative emotions to explore these effects. Using data from the 2020 CHARLS data, it examines how different forms of intergenerational support affect the mental health of the older adults. The paper also conducts heterogeneity analysis based on household registration, health status, gender, and income levels.

## 3 Materials and methods

### 3.1 Theoretical analysis and research hypotheses

#### 3.1.1 Theoretical analysis

Intergenerational support from adult children, as a primary form of informal social support, can be understood through the lens of social support theory. Scholars have reached a preliminary consensus on the role of social support in mental health, primarily identifying two models: the main effect model and the buffering model (43). The main effect model focuses on the enhancement of positive emotions in the care recipients, while the buffering model emphasizes the alleviation of negative emotions. The main effect model holds that social support can generally enhance the physical and mental health of individuals by maintaining their good emotional experience and physical and mental condition (43, 44). The hypothesis of the main effect of intergenerational support on the mental health of the older adults has been verified in many empirical studies (7, 22). The buffer model holds that social support can alleviate the negative emotions of individuals in the face of stressful events, thus protecting the mental health of individuals (43, 45). Some empirical studies have confirmed the buffer model, adult children’s financial support can significantly reduce depression symptoms of older adult parents (46), and emotional support can alleviate depression or loneliness in older people (14, 28).

Social exchange theory is also often used to explain intergenerational support. From the perspective of social exchange, when the older adults have poor resources and cannot establish a reciprocal and balanced exchange relationship, they will feel disconnected from society (10, 47). If this unequal exchange relationship is further exacerbated, for example, the older adults need long-term support from their adult children because of long-term disability or constant stress, it may also bring a special negative emotion - shame. Shame is a concept in social psychology related to emotional expression. It is triggered by devaluation and serves as an adaptive signal to the self, warning of diminished social rank (48–50). The phenomenology of shame includes feelings of being weak, small, inhibited, and confused (51). When parents find themselves dependent on their adult children for daily care, the devaluation due to their physical and mental weakness generates shame, potentially lowering their mental health.

Additionally, the inherent altruism of parents toward their adult children may cause the older adults to feel uneasy and guilty when receiving care, especially among disabled older individuals. The buffering effect of intergenerational support on the mental health of disabled older adults may thus be negative. This might be due to the cultural norm of “responsibility ethics” in older adults’ care in China, where the older people emphasize their altruistic contributions to their adult children and are reluctant to become a burden in their old age (16). Therefore, adult children’s caregiving, especially physical care, can lead to feelings of shame and guilt in the older adults, generating new negative emotions. In recent years, there have been instances of rural older individuals choosing to commit suicide when they become purely burdens to their families due to illness or disability, with strong intergenerational responsibility serving as the ethical support for such altruistic suicides (52).

Social relationships and social participation are also important dimensions influencing mental health. The Social Convoy Model shapes the relationship between individuals and society into three concentric circles: inner, middle, and outer circles, explaining how interactions in the inner circle serve as the foundation for social participation in the outer circle. For older individuals, the social relationships within the inner circle, particularly family support, are the most crucial. Family support can reduce external pressures and strengthen the connection between older adults and society, thereby promoting social participation (36–38). The Social Contagion Theory emphasizes that older individuals can reduce social isolation through social participation. A “group interaction” environment and the corresponding positive social psychological processes help prevent older adults from experiencing social exclusion and enhance social integration, thereby mitigating feelings of loneliness (15, 53, 54). When both the social convoy model and social contagion theory operate simultaneously, family support may influence the mental health of older individuals by affecting their social participation, creating a mediating effect.

#### 3.1.2 Research hypotheses

Based on the main effect model, economic support and caregiving from adult children can improve the mental health of the older adults. On one hand, after retirement, reduced income means that older individuals can only meet basic living needs, especially those with illnesses. Intergenerational economic support from adult children can improve their living conditions, allowing them to access better medical resources or pursue higher-level spiritual needs, thereby enhancing their mental health. On the other hand, as older individuals’ social relationships and networks shrink with age, losing potential social support from colleagues and friends, adult children’s support becomes dominant in their social support network. Moreover, older individuals have high expectations for filial piety from their adult children. When adult children provide economic support and caregiving, it not only meets the older adults’ expectations but also instills a sense of achievement in parenting, further benefiting their mental health.

*Hypothesis 1*: Intergenerational economic support from adult children enhances the mental health of older individuals by boosting their positive emotions.

*Hypothesis 2*: Caregiving support from adult children enhances the mental health of older individuals by boosting their positive emotions.

The effects of different family support on mental health of the older adults are different. He et al. (55) empirical study on older adults in China found that, different from the positive effects of economic support and emotional support, the life care or service support provided by adult children to their parents did not affect the mental health of the older people. Furthermore, receiving domestic support from adult children is detrimental to the mental health of older persons (19, 56). These empirical findings lead us to ask why adult children’s life care and support for their parents have different effects on mental health.

Unlike financial support or emotional comfort, life care often occurs when the older adults are in poor health or even disabled. When adult children care for disabled older adult parents, the emotional, cost, and economic support required is greater than for non-disabled older people, potentially causing feelings of guilt in the disabled older people. The stronger the guilt in severely disabled younger older adults, the stronger the depressive emotions (57).

Due to the simultaneous presence of satisfaction from receiving care and guilt towards the adult children, the impact mechanism of caregiving on the older adults’ mental health becomes complex, and the ultimate direction of this impact may be uncertain.

*Hypothesis 3*: Caregiving support from adult children reduces the mental health of older individuals by triggering negative emotions.

Drawing upon the buffering model, it can be posited that financial support from adult children plays a crucial role in aiding older individuals overcome hardships, thereby reducing negative emotional states. Additionally, emotional support from adult children can alleviate the negative emotions arising from self-perceptions of aging. As older individuals grow old, their physiological functions decline, cognitive abilities decrease, and they face increased life stress, leading to feelings of loneliness and helplessness, which in turn increase negative emotions and even depression. Emotional support from adult children lays a pivotal role in mitigating feelings of loneliness, eliminating negative emotions, and improving mental health.

*Hypothesis 4*: Intergenerational financial support from adult children contributes to the enhancement of the mental health of older individuals by reducing their negative emotions.

Hypothesis *5*: Emotional support from adult children contributes to the enhancement of the mental health of older individuals by reducing their negative emotions.

More social participation of the older adults can not only ensure that the older people stay in society and meet the basic needs of social communication, but also ensure the cognitive ability of the older people and relieve mental pressure. Therefore, participation in social activities can promote the mental health of the older adults. Economic conditions are an important factor affecting social participation (15). The content and form of social activity participation are constrained by economic resources. Intergenerational economic support from adult children can improve the economic conditions of the older adults, and the positive feedback of adult children’s social norms of “filial piety” can enhance the older people’s willingness to participate in social activities, and their participation frequency will be higher. Participation in social activities can significantly reduce depression levels or suppress loneliness in older adults (14, 15).

This paper attempts to link the above economic support effect with the health promotion effect of social participation, and argues that adult children’s financial support can improve the mental health of the older adults through enhancing social participation. Therefore, the last hypothesis of this paper is proposed:

*Hypothesis 6*: Social participation mediates the relationship between intergenerational economic support and older adults’ mental health, with intergenerational economic support promoting social participation and thereby enhancing mental health.

### 3.2 Research design

#### 3.2.1 Data source

The data for this study were derived from the 2020 wave of the China Health and Retirement Longitudinal Study (CHARLS). CHARLS dataset covers 32 provinces (autonomous regions and municipalities) across China, employing a multistage probability proportional to size (PPS) sampling method, resulting in a total sample size of 19,395 respondents.

First of all, according to the research requirements of this paper, we need to merge the personal information database, family member information database and family income database of CHARLS in 2020, using personal ID, household ID and community ID to identify and match. The total number of samples after merger is 19,331. Secondly, to maximize the retention of sample information, missing values for the explanatory variables of interest in this study are handled by assigning them a value representing the lowest level, commonly referred to as “none,” or similar terms. This approach treats these cases as part of the control group. Finally, to define our population of study, we selected the older adults aged 60 years and above as the research objects, and finally obtained 10,620 valid samples through age screening. To ensure the absence of multicollinearity in our sample, we conducted a collinearity analysis of the variables. The results indicated that all variables had Variance Inflation Factor (VIF) values below 10, with an overall VIF coefficient of 1.23, indicating no multicollinearity issues. Meanwhile, principal component analysis (PCA) results showed that the first principal component explained 34.24% of the variance (<50%), and no significant common method bias was detected.

#### 3.2.2 Variable settings and descriptive statistical analysis

The main explained variable in this paper was the mental health level of the older adults, which was calculated based on the simplified CES-D scale provided by CHARLS data by referring to the practice of Mu and Yuan (58) and Zhang and Li (59). We used the main effect model and buffer model to explain the mechanism of intergenerational support on the mental health of the older adults. With reference to the research of Radloff (60) and Li and Wu (61), this paper extracted two main factors of positive emotion and negative emotion for 10 items of the simplified scale, as the mental health results of two dimensions of main effect and stress relief effect. Responses to the scale items were rated from 1 to 5. Negative emotion scores were reverse-coded and then combined with positive emotion scores to yield an overall mental health score.

The core explanatory variables are adult children’s economic support, caregiving, and emotional support. The existing literature has a variety of ways to measure financial support, some scholars set whether to provide support dummy variables (14, 62), and more studies directly use the monetary value of money provided by adult children and physical goods (12, 19, 22, 42) or grouping the monetary value of economic support (55).

Adult children’s emotional comfort support for the older adults was generally measured by whether dummy variables were provided (14, 15, 62), the frequency of meeting or contacting with adult children or frequently-based categorical variables (28, 29, 55), the total time spent with adult children (12) or the score of emotional intimacy (19, 36).

There are many ways to measure adult children’s care support for the older adults. They include whether in-person care is provided (14, 62), the cumulative time of daily care assistance, the frequency of in-person care provided, or a categorical variable based on frequency (19, 55, 63).

Building on previous literature, this paper compares the assignment experience of the variables supported by the above generations, and selects the assignment methods based on the inter-generation support frequency. According to the content of the CHARLS2020 questionnaire, the measurement question of financial support is set to “How much financial support (including in-kind conversion) did your adult children give you in the past year?” To eliminate outliers, this paper takes the logarithm of financial support.

The measurement question of daily care is set as “who helps you in the above difficulties (dressing, bathing, eating, getting up, going to the toilet, housework, cooking, shopping, making phone calls, taking medicine, managing money, etc.),” and the approach adopted by Wei et al. (14) and Wu and Jia (62) is treated as a binary categorical variable in this paper. That is, if the answer is “adult children, daughter-in-law, son-in-law, grandchildren,” it is considered that the adult children have provided care.

The measure of emotional comfort was set to “How often do you see your adult children when they are not living together?” CHARLS2020 questionnaire divides the frequency of meeting into 9 types. To make the score and emotional comfort support show positive changes, this study conducted reverse assignment according to the content of the questionnaire, that is, 0 represents “almost never” and 8 represents “almost every day.” At the same time, in order to reflect the influence of the number of adult children, this study summed up the scores of emotional comfort provided by different adult children.

Our models control for a range of personal characteristics, socioeconomic circumstances and health characteristics. Personal characteristics included gender, age, education level, and marital status. Socioeconomic characteristics included household registration status and annual income. Health characteristics included chronic disease status, disability status, and self-rated health.

The mediating variable, social activity participation, is derived from the question “Have you engaged in any of the following social activities in the past month (multiple choices are available), including: (1) visiting, socializing with friends; (2) Play mahjong, chess, cards, go to the community room; (3) Providing assistance to relatives, friends or neighbors who do not live with you; (4) Dancing, fitness, Qigong, etc.; (5) Participate in activities organized by associations; (6) Volunteer activities, or charity activities, or care for sick or disabled people who do not live with you; (7) Attending school or training courses; (8) Other social activities. Our assignment also considered further inquiries about social activities, “In the past month, how often did you engage in these activities?” Responses to this question were coded as: almost daily (3 points), almost weekly (2 points), infrequently (1 point), and not at all (0 points). Social activity participation is measured constructed by aggregating the scores of various activities to obtain a comprehensive social participation score for the older adults.

The specific variable definitions are provided in Table 1.

**Table 1:** Descriptive statistics of variables.

Variable	Value assignment	Mean	Min	Max
Mental health	Continuous variable, positive emotion assigned forward, negative emotion assigned backward, then added	29.50	10	40
Positive emotion	Continuous variable, total value of positive emotion questions in CES-D scale	5.42	2	8
Negative emotion	Continuous variable, total value of negative emotion questions in CES-D scale	15.92	8	32
Financial support	Continuous variable	6.95	0	13.06
Daily care	Yes = 1, No = 0	0.15	0	1
Emotional comfort	Continuous variable	9.37	0	54
Gender	Male = 1, Female = 0	0.48	0	1
Age	Continuous variable	68.98	60	108
Education level	Illiterate or Did Not Complete Primary School =0, Graduated From Private School or Primary School = 1, Junior High = 2, High School and above = 3	0.97	0	3
Marital status	Married = 1, Other = 0	0.76	0	1
Household registration	Urban = 1, Rural = 0	0.27	0	1
Annual income (ln)	Continuous variable	7.27	0	13.25
Daily activity ability (ADL)	Disabled = 1, Not disabled = 0	0.43	0	1
Chronic diseases	Yes = 1, No = 0	0.42	0	1
Self-rated health	Very good = 5, Good = 4, Average = 3, Bad = 2, Very bad = 1	2.96	1	5
Pension income(ln)	Continuous variable	6.35	0	13.25
Social activity participation	Continuous variable, total value based on participation frequency in various activities	1.22	0	14

#### 3.2.3 Quantitative model

Drawing on the health demand model, this study examines the impact of intergenerational support from adult children (economic support, caregiving, and emotional comfort) on the mental health of the older adults. The dependent variable is mental health of older adults, with the three types of intergenerational support as independent variables. Control variables include demographic characteristics, socioeconomic characteristics, and physical health status. An OLS benchmark model is constructed as follows:


(1) $$\begin{array}{l} \mathrm{Menta}l_{-}\mathrm{health}=\beta_{1}•\mathrm{Money}+\beta_{2}•\mathrm{Care}+\beta_{3}•\mathrm{Emotion}\\ +\beta_{4}•\mathrm{Control}+\varepsilon \end{array}$$

Where *Mental health* is explained variable, representing the mental health level of the older adults. Money, Care, Emotion represent adult children’s financial support, daily care, and emotional comfort from adult children, respectively. *Control* represents control variables including demographic, socioeconomic, and health status of the old persons, and ε is the error term.

To address potential endogeneity arising from the bidirectional relationship between mental health and intergenerational support of older adults, we will use the instrumental variable method to solve the possible endogeneity problems. In the robustness test, the interpreted variable was replaced by the multi-categorical ordered variable of life satisfaction to observe the reliability of the results. Heterogeneity analysis will be conducted from multiple aspects.

## 4 Results

### 4.1 Results of the multiple linear regression model

Table 2 reports the regression results of intergenerational support on the mental health of the older adults. Model 1 includes only control variables such as age and gender. Models 2, 3, and 4 sequentially add intergenerational support variables, namely economic support, daily care, and emotional comfort, respectively. While Model 5 includes all intergenerational support variables along with control variables.

**Table 2:** Regression results of adult children’s intergenerational support on the mental health of the older adults.

Variables	Model 1	Model 2	Model 3	Model 4	Model 5
Financial support		0.087*** (4.66)			0.065*** (3.30)
Daily care			−1.027*** (−5.50)		−1.028*** (−5.50)
Emotional comfort				0.033*** (3.95)	0.025*** (2.83)
Gender	1.029*** (8.50)	1.051*** (8.68)	0.995*** (8.21)	1.064*** (8.77)	1.038*** (8.55)
Age	−0.048*** (−5.35)	−0.053*** (−5.86)	−0.041*** (−4.50)	−0.062*** (−6.44)	−0.055*** (−5.68)
Education level	0.278*** (4.92)	0.280*** (4.97)	0.275*** (4.89)	0.289*** (5.13)	0.286*** (5.08)
Marital status	1.174*** (8.10)	1.096*** (7.51)	1.090*** (7.48)	1.136*** (7.82)	1.002*** (6.84)
Household registration	1.043*** (7.03)	1.102*** (7.41)	1.049*** (7.08)	1.082*** (7.28)	1.123*** (7.56)
Income	0.112*** (3.83)	0.115*** (3.92)	0.112*** (3.84)	0.113*** (3.87)	0.115*** (3.94)
Chronic diseases	−0.719*** (−6.07)	−0.727*** (−6.14)	−0.698*** (−5.89)	−0.722*** (−6.09)	−0.705*** (−5.96)
ADL	−3.206*** (−25.12)	−3.202*** (−25.12)	−2.896*** (−20.77)	−3.207*** (−25.15)	−2.893*** (−20.77)
Self-rated health	1.724*** (26.85)	1.726*** (26.92)	1.724*** (26.89)	1.719*** (26.79)	1.723*** (26.90)
Pension income(ln)	0.056** (2.21)	0.051** (2.03)	0.054** (2.14)	0.055** (2.16)	0.050* (1.95)
Constant	26.310*** (37.84)	26.079*** (37.45)	25.908*** (37.11)	26.980*** (37.72)	26.243*** (36.19)
N	10,620	10,620	10,620	10,620	10,620
R-squared	0.246	0.248	0.248	0.247	0.250

***, **, * indicate significance at 1, 5, and 10% levels, respectively; *t*-values are in parentheses.

Model 1 reflects the impact of control variables on older adults’ mental health. Model 2 adds financial support to Model 1. Regression results showed that when the original hypothesis of no correlation between financial support and mental health of the older adults was rejected at the significance level of 1%, the mental health score of the older people increased by 0.00087 when the financial support of the adult children increased by 1%. Adult children’s financial support can alleviate the economic pressure of the older people, improving their quality of life. The behavior aligns with the traditional Chinese culture of “filial piety,” meeting the psychological expectations of the older people and thus improving their mental health.

Model 3 adds daily care variable to Model 1, showing a significantly negative impact of adult children’s daily care on the mental health of the older adults. The reverse effect of adult children’s life care on the mental health of the older adults is worthy of further discussion. It is the comprehensive result caused by positive emotions and negative emotions.

Model 4 adds emotional comfort variable to Model 1. Similar to the findings of the study by He et al. (55), emotional support improves mental health of older adults.

Model 5 also examines the combined effects of three types of intergenerational support-economic support, daily care and emotional comfort-on the mental health level of the older adults, and the findings remains consistent with the previous models. The regression model is established based on Equation 1.

### 4.2 Impact on positive and negative emotions

To explore the impact mechanism of different forms of adult children’s intergenerational support on the mental health of the older adults, this study constructs measurement models based on positive and negative emotions of the older adults to examine how different types of intergenerational support affect the mental health of the older people. The specific model settings are as follows:


(2) $$\begin{array}{l} \mathrm{Positive}=\alpha_{1}•\mathrm{Money}+\alpha_{2}•\mathrm{Care}+\alpha_{3}•\mathrm{Emotion}\\ +\alpha_{4}•\mathrm{Control}+\varepsilon \end{array}$$

(3) $$\begin{array}{l} Neg\mathrm{ative}=\gamma_{1}•\mathrm{Money}+\gamma_{2}•\mathrm{Care}+\gamma_{1}•\mathrm{Emotion}\\ +\gamma_{4}•\mathrm{Control}+\varepsilon \end{array}$$

Table 3 reports the regression results of adult children’s intergenerational support on the positive emotions of the older adults. Model 2, Model 3 and Model 4 show that the coefficients of the three types of support reject the null hypothesis that they have no effect on the positive emotions of the older adults at the significance level of 1%, that is, the three types of support all enhance the positive emotions of the older people. However, when all three types of intergenerational support are considered, the effect of emotional comfort is not statistically significant. Model 5 presents the regression results of Equation 2, indicating that a 1% increase in adult children’s financial support for the older adults raises older people’s positive emotion score by 0.00026. Additionally, daily care provided by adult children increased positive emotion scores by an average of 0.317 points. This result is consistent with hypothesis 1 and hypothesis 2 above. The effect of emotional comfort on positive emotions of older adults is not significant. This may be because that when older individuals receive emotional comfort support from their adult children, they tend to relieve their own depression and eliminate loneliness, which has little impact on positive emotions.

**Table 3:** Regression results of adult children’s intergenerational support on positive emotions of the older adults.

Variables	Model 1	Model 2	Model 3	Model 4	Model 5
Financial support		0.028*** (4.85)			0.026*** (4.22)
Daily care			0.317*** (5.40)		0.321*** (5.48)
Emotional Comfort				0.009*** (3.34)	0.004 (1.56)
Control	–	Controlled			
Constant	3.960*** (18.16)	3.885*** (17.78)	4.084*** (18.65)	4.138*** (18.44)	4.105*** (18.04)
N	10,620	10,620	10,620	10,620	10,620
R-squared	0.066	0.068	0.069	0.067	0.071

***, **, * indicate significance at 1, 5, and 10% levels, respectively; *t*-values are in parentheses.

The results in Table 4 show that adult children’s financial support and emotional comfort significantly reduce the negative emotions of the older adults, but daily care has a significantly positive effect on the negative emotions of the older people. This result is consistent with hypothesis 3, 4, and 5. Model 5 represents the estimation results of Equation 3.

**Table 4:** Regression results of adult children’s intergenerational support on negative emotions of the older adults.

Variables	Model 1	Model 2	Model 3	Model 4	Model 5
Financial support		−0.059*** (−3.26)			−0.039** (−2.05)
Daily Care			1.344*** (7.48)		1.350*** (7.50)
Emotional Comfort				−0.024*** (−3.01)	−0.021*** (−2.44)
Control	–	Controlled			
Constant	17.650*** (26.38)	17.806*** (26.55)	18.176*** (27.08)	17.159*** (24.92)	17.861*** (25.61)
N	10,620	10,620	10,620	10,620	10,620
R-squared	0.212	0.213	0.217	0.213	0.217

***, **, * indicate significance at 1, 5, and 10% levels, respectively; *t*-values are in parentheses.

### 4.3 Mediation effect of social activity participation

Adult children’s financial support improves the economic conditions of the older adults, providing a material basis for their participation in social activities. Higher income may encourage older adults to participate more frequently in social activities. The social convoy model suggests that family support can alleviate external pressure and strengthen the connection between the older adults and society, thus promoting social participation. In addition, the social contagion theory further emphasizes that social participation is a positive social psychological process, and the involvement in social activities can inhibit social loneliness. It can alleviate depression and loneliness and other negative emotions, and improve the mental health level of the older adults. To verify whether social activity participation mediates the relationship between adult children’s financial support and the mental health of the older adults, a mediating effect test is conducted. Based on the stepwise regression test procedure for mediating effects proposed by Wen et al. (64), the following stepwise regression models are established (Equations 4-6):


(4) $$\begin{array}{l} Mental_{-}\mathrm{health}=\beta_{1}•\mathrm{Money}+\beta_{2}•\mathrm{Care}+\beta_{3}•\mathrm{Emotion}\\ +\beta_{4}•\mathrm{Control}+\phi \end{array}$$

(5) $$\begin{array}{l} Ac\mathrm{tivity}=\delta_{1}•\mathrm{Money}+\delta_{2}•\mathrm{Care}+\delta_{3}•\mathrm{Emotion}\\ +\delta_{4}•\mathrm{Control}+\phi \end{array}$$

(6) $$\begin{array}{l} \mathrm{Menta}l_{-}\mathrm{health}=\xi_{1}•\mathrm{Money}+\xi_{2}•\mathrm{Activity}+\xi_{3}•\mathrm{Care}+\xi_{4}\\ •\mathrm{Emotion}+\xi_{5}•\mathrm{Control}+\phi \end{array}$$

The regression results are presented in Table 5 below: Model 1 is the regression results of adult children’s financial support on the mental health of older adults, and intergenerational financial support improves the mental health of older individuals; Model 2 is the regression result of adult children’s financial support on the older adults’ participation in social activities, adult children’s financial support promotes social participation, the empirical result supports the theory put forward by the social convoy model, that is, family support can promote social participation; Model 3 also considers the variables controlling for adult children’s economic support and social activity participation. The results show that both variables contribute to mental health, and social activity participation plays a partial mediating role. Hypothesis 6 is valid and this empirical result should also support the theory put forward by social contagion theory, which suggests that social contagion can improve the mental health of the older adults. After adding the mediating variable of social activity participation, the economic support coefficient in Model 3 decreases compared to model 1, which may be because some of the mental health needs of the older adults are met in social activity participation, which reduces older people’s reliance on financial support from their adult children, indicating that social activity participation may have a substitutive effect on economic support.

**Table 5:** Regression analysis of the mediating effect of social activity participation.

Variables	Model 1 (Dependent variable: mental health)	Model 2 (Dependent variable: social activity participation)	Model 3 (Dependent variable: mental health)
Financial support	0.065*** (3.30)	0.040*** (6.63)	0.057*** (2.91)
Social activity participation	–	–	0.197*** (6.15)
Daily Care	−1.028*** (−5.50)	−0.207*** (−3.65)	−0.988*** (−5.29)
Emotional Comfort	0.025*** (2.83)	0.001 (0.35)	0.025*** (2.82)
Control	Controlled		
Constant	26.244*** (36.19)	1.569*** (7.14)	25.935*** (35.74)
N	10,620	10,620	10,620
R-squared	0.250	0.049	0.253

***, **, * indicate significance at 1, 5, and 10% levels, respectively; *t*-values are in parentheses.

To accurately test the mediating effect, the Bootstrap method is used for verification, with an effect size of 0.010 (Boot SE = 0.003, 95% CI [0.005, 0.015]).The Bootstrap verification results show that social activity participation has a partial mediating effect on the impact of adult children’s financial support on the mental health of the older adults, validating hypothesis 6. This indicates that adult children’s financial support can enhance the older adults’ economic capacity for social participation, promoting more social activities, and thereby improving mental health.

### 4.4 Endogeneity test

The potential endogeneity of the impact of intergenerational support from adult children on older adults’ mental health may stem from two sources. First, there might be a reciprocal causal relationship between older adults mental health levels and intergenerational support behaviors. For example, the provision of intergenerational support by adult children could be a response to a decline in the older adults’ mental health; thus, when an older people person experiences poor mental health, their adult children may offer more support. Second, omitted variable bias may arise from unobservable factors, such as varying perceptions of the need for intergenerational support among the older adults.

To address potential endogeneity issues, this study employs an instrumental variable (IV) approach using the IV-OLS model, with the results presented in Tables 6–8. Instrumental variables must be correlated with the core explanatory variables and uncorrelated with the random error terms, ensuring their exogeneity. Following the method of Chyi and Mao (65) this study selects the “income level of the eldest son or daughter” as an instrument for intergenerational support. The rationale is that the eldest son or daughter often bears a significant share of the responsibility for supporting older adult parents, with their income level affecting the economic support that older individuals receive, but not directly influencing the older people’s mental health.

**Table 6:** Regression results of intergenerational support on older adults life satisfaction.

	Variables	Model 1	Model 2	Model 3	Model 4
Financial support		0.011*** (4.89)			0.010*** (3.94)
Daily care			0.023 (0.97)		0.023 (1.00)
Emotional comfort				0.004*** (3.67)	0.002** (2.12)
Control	–	Controlled (Mediate variable is involved)			
N	10,620	10,620	10,620	10,620	10,620
R-squared	0.077	0.079	0.077	0.078	0.079

***, **, * indicate significance at 1, 5, and 10% levels, respectively; *t*-values are in parentheses.

**Table 7:** Regression results of redefined daily care on older adults mental health.

	Model 1	Model 2	Model 3	Model 4
Financial support		0.079*** (4.21)	0.071*** (3.82)	0.059*** (2.98)
Daily care			0.822*** (7.05)	0.802*** (6.85)
Emotional comfort				0.017* (1.92)
Control	–	Controlled (Mediate variable is involved)		
Constant	25.942*** (37.27)	25.749*** (36.94)	25.571*** (36.74)	25.954*** (35.86)
N	10,620	10,620	10,620	10,620
R-squared	0.249	0.250	0.254	0.254

***, **, * indicate significance at 1, 5, and 10% levels, respectively; *t*-values are in parentheses.

**Table 8:** Regression results of intergenerational support from adult children for older adults with different household registrations.

	Mental health		Positive emotion		Depression emotion	
	Urban	Rural	Urban	Rural	Urban	Rural
Financial support	0.097*** (2.80)	0.039 (1.59)	0.031*** (2.92)	0.020*** (2.67)	−0.065** (−2.03)	−0.018 (−0.77)
Daily care	−1.651*** (−4.16)	−0.797*** (−3.76)	0.325*** (2.64)	0.344*** (5.16)	1.976*** (5.35)	1.141*** (5.52)
Emotional comfort	0.030 (1.56)	0.024** (2.33)	0.004 (0.70)	0.005 (1.42)	−0.026 (−1.44)	−0.019** (−1.93)
Control	Controlled (Mediate variable is involved)					
Constant	27.444*** (20.13)	25.736*** (29.64)	4.477*** (10.57)	3.907*** (14.33)	17.033*** (13.42)	18.171*** (21.49)
N	2,831	7,789	2,831	7,789	2,831	7,789
R-squared	0.246	0.232	0.102	0.045	0.212	0.206

***, **, * indicate significance at 1, 5, and 10% levels, respectively; *t*-values are in parentheses.

The method of using the economic status or social support variable with a one-stage lag as the instrumental variable has been used many times (66). This paper adopts a similar approach to the handling of the two endogenous explanatory variables of life care and emotional comfort, respectively using the score of adult children’s life care and emotional comfort with a one-stage lag as the instrumental variable. In terms of exogeneity, past emotional support from adult children does not directly affect the current mental health of the older adults, whereas past support influences the frequency of current emotional support received.

The first-stage regression results reveal that the instrumental variables in all three models are statistically significant at the 1% level, Moreover, the F-statistics from the first stage of regression are all greater than 10, rejecting the hypothesis of weak instruments, and the model estimates are reliable.

Model 1 in Table 9 presents the IV-OLS regression results for the impact of economic support on older adults mental health, demonstrating a significant positive effect. Model 2 reports the IV-OLS regression results for the effect of adult children’s daily care on older adults’ mental health, indicating a significant negative effect. Model 3 reveals the IV-OLS regression results for the impact of emotional support on older adults’ mental health, demonstrating a significant positive effect of emotional support on mental health. The regression results from all three models are consistent with the baseline regression findings, and the estimates for the control variables align with expectations, confirming the robustness of the baseline regression.

**Table 9:** IV-OLS regression results for intergenerational support.

IV Model	IV-OLS		N	Weak instrument test
	First stage (Dependent variable: IV)	Second stage (Dependent variable: Mental health)		
Model 1: Economic support (IV: the economic income level of the eldest son or eldest daughter)	0.165*** (−16.15)	0.488*** (−4.12)	10,620	*F* = 260.93
Model 2: Life care (IV: delayed life care)	0.232*** (−17.93)	−5.041*** (−6.00)	9,736	*F* = 321.54
Model 3: Emotional comfort (IV: one period of delayed emotional comfort)	0.698*** (−63.47)	0.046*** (−3.16)	5,999	*F* = 4028.97

***, **, * indicate significance at 1, 5, and 10% levels, respectively; *t*-values are in parentheses.

### 4.5 Robustness check

Life satisfaction is one of the most commonly used measures of cognitive life evaluations (23). To verify the reliability of the aforementioned regressions, this paper adopts the approach of Wu (23) and Cheng (67), substituting the variable of life satisfaction for mental health level in the regressions, thereby enhancing the credibility of the conclusions. The life satisfaction variable is defined based on responses to the CHARLS questionnaire item, “Overall, are you satisfied with your life?” Responses are assigned values from 5 to 1. Given that life satisfaction is an ordered categorical variable, an Ordered Logistic model is employed for the regressions. Considering the variables set in the previous sections, the model for older adults life satisfaction is established as follows:


(7) $$\begin{array}{l} \log\left(\mathrm{satisfaction}\right)=\theta_{k}+\eta_{1}•\mathrm{Money}+\eta_{2}•\mathrm{Care}+\eta_{3}\\ •\mathrm{Emotion}+\eta_{4}•\mathrm{Control}+\varepsilon \end{array}$$

Where Money, Care and Emotion represent intergenerational economic support, daily care, and emotional comfort from adult children, respectively, and Control denotes a series of control variables.

Table 6 reports the regression results of intergenerational support from adult children on the life satisfaction of the older adults’, as outlined in Equation 7. The results indicate that a 1% increase in adult children’s economic support raises the life satisfaction of the older adults by 0.00011, and a 1-point increase in adult children’s emotional support score raises the life satisfaction of the older adults by 0.023. The impact of daily care support is not significant, which is consistent with the previous regression results.

Given that older adults tend not to require daily care from their adult children if they are capable of self-care, the daily care variable is redefined to encompass both current and anticipated future care. This redefinition is based on the relevant items from CHARLS questionnaire: (1) “Who helps you the most with difficulties such as dressing, bathing, eating, getting up, toileting, housework, cooking, shopping, making phone calls, taking medication, and managing money?” and (2) “If you need care in daily life in the future, who will take care of you?” If either response indicates adult children as caregivers, the variable is assigned a value of 1; otherwise, it is assigned a value of 0.

As shown in Table 7, when the daily care variable is defined to include both current and anticipated future care from adult children, the effect on the mental health of the older adults becomes significantly positive. This may be because, even when older individuals are physically healthy and capable of normal daily activities, nearly half of them expect to receive care from their adult children in case of future illness or incapacity. This positive expectation can significantly enhance their mental health.

### 4.6 Heterogeneity analysis

#### 4.6.1 Urban–rural differences

There is a significant disparity in development levels between urban and rural areas in China. Many rural youth migrate to cities for work, resulting in a large number of left-behind older adults in rural areas. These rural older adults, who are often unable to see their adult children for extended periods, may have unmet emotional needs. Consequently, rural older adults might have a higher demand for emotional comfort compared to their urban counterparts. There are significant disparities between urban and rural areas in China in terms of economic development levels, work practices, and living and lifestyle patterns. Therefore, this paper categorizes the older adults into urban and rural groups based on their household registration to explore whether the effects of different forms of intergenerational support on the mental health of the older adults differ between urban and rural areas.

The regression results are shown in Table 8. Financial support from adult children has a significant positive impact on the positive emotions of urban older individuals, a significant negative impact on negative emotions, and a significant positive effect on overall mental health. In contrast, while economic support from adult children significantly enhances the positive emotions of rural older individuals, its impact on their mental health and negative emotions is not significant. This finding is consistent with that of Hou et al. (42), who did not provide an explanation for it. Generally, in more traditionally cultural regions, the impact of adult children’s economic support on the mental health of the older adults is more pronounced. For example, adult children’s economic support significantly increased the life satisfaction of older individuals in South Korea, but this effect was not observed in the United States (40). This result does not fully align with general trends.

A possible explanation is that adult children’s economic support may enhance positive emotions through the positive feedback of “filial piety,” which applies to both urban and rural older adults. However, urban older individuals tend to have higher living expenses and more diverse needs, making low-income individuals more susceptible to negative emotions stemming from financial strain. Therefore, economic support from adult children can improve their situation and alleviate negative psychological states. In contrast, rural older individuals have a higher degree of self-sufficiency in their daily lives and face a less diverse market, so the economic support has limited effects on alleviating negative emotions.

Life care has a significant negative impact on the overall mental health of older individuals, but it has a significant positive impact on the positive emotions of both rural and urban older adults. This indicates that older individuals receiving life care experience a certain degree of psychological comfort, which can somewhat enhance their mental health levels. The impact of life care on negative emotions is more pronounced among urban older individuals, possibly because urban older adults have weaker traditional views on aging or possess stronger self-esteem, making them more susceptible to feelings of guilt triggered by adult children’s life care.

The impact of emotional support on the negative emotions and mental health of rural older individuals rejects the null hypothesis at the 5% level, showing both negative and positive effects. However, emotional support has no significant effect on the positive emotions, negative emotions, or mental health levels of urban older individuals. This result is generally consistent with the findings of Tang et al. (41), possibly because urban older individuals rely more on extensive social networks. In contrast, rural older individuals are often left-behind seniors, with their adult children away for long periods, resulting in a higher degree of emotional need for their adult children. Thus, they may have a greater expectation for emotional support and comfort from their children.

#### 4.6.2 Health status differences

Older individuals with varying health statuses have different demands for various forms of intergenerational support. Compared to non-disabled older adults, disabled older people, confined to their homes due to long-term disability, may find that economic support from adult children does little to improve their quality of life, while emotional comfort might better meet their internal needs and enhance their mental health. Most empirical studies often control for the physical health status of older individuals when estimating the impact of family support or social support on their mental health (7, 67, 68). Both external support and health jointly influence the mental health of older adults. Lafferty and Phillips (69) based on data from Ireland, found that individuals in poor health derive greater mental health benefits from social support. Similarly, an empirical study by Shi et al. (70) in China found that external support moderates the negative impact of physical disabilities on the mental health of older individuals. We believe that the demand for the three types of support among disabled older individuals changes, with a greater need for life care and a higher likelihood of experiencing negative emotions. Therefore, discussing the impact of intergenerational support on mental health by distinguishing between disabled and non-disabled groups is more valuable. Additionally, research focused on disabled individuals can reveal the comprehensive mechanisms of care support on mental health across two dimensions: the positive emotions derived from the fulfillment of “filial piety” and the negative feelings of guilt stemming from being a burden to their adult children.

The regression results are shown in Table 10 above. For disabled older adults, adult children’s financial support has a positive effect on their emotions and mental health and the null hypothesis is rejected at the significance level of 1 and 10%, indicating a positive effect. However, intergenerational financial support cannot improve the negative emotions of disabled older adults, which is similar to the results obtained by Sun et al. (71) and Hu and Liu (72).The emotional comfort of the disabled older adults is conducive to mental health. However, there are differences in the conclusions on life care. Hu and Liu (72) found that long-term caregiving by family members improves the mental health of disabled older adult individuals, whereas this study finds that while caregiving support enhances their positive emotions, it also exacerbates their negative emotions. Additionally, our findings are similar to those of Li et al. (73), persons with disabilities who have received long-term family care generally have serious psychological burden. For non-disabled older adults, adult children’s financial support has a significant positive impact on their positive emotions, a significant negative impact on their negative emotions, and a significant positive impact on their overall mental health level. This is because the financial support provided by adult children enables non-disabled older adults to achieve a better standard of living, leading to greater participation in social activities and the pursuit of higher-level needs, thereby enhancing positive emotions, reducing negative emotions, and improving overall mental health. Emotional comfort has a significant negative impact on negative emotions and a significant positive impact on mental health levels for the older adults, though the significance level is lower. This may be because non-disabled older people have a higher probability of participating in social activities and have more diverse sources for meeting their emotional needs, thereby reducing their dependence on emotional support from their adult children.

**Table 10:** Regression results of intergenerational support from adult children for older adults with different health statuses.

	Mental health		Positive emotion		Depression emotion	
	Disabled	Nondisabled	Disabled	Nondisabled	Disabled	Nondisabled
	0.061* (1.84)	0.053** (2.19)	0.026*** (2.68)	0.022*** (2.72)	−0.035 (−1.08)	−0.031 (−1.36)
Financial Support	−1.028*** (−5.12)		0.289*** (4.96)		1.317*** (6.73)	
Daily care	0.022* (1.66)	0.027** (2.18)	0.006 (1.46)	0.003 (0.80)	−0.016 (−1.27)	−0.023** (−2.01)
Emotional Comfort	Controlled (Mediate variable is involved)					
Control	23.115*** (21.73)	25.718*** (25.57)	3.203*** (10.36)	4.574*** (13.71)	20.088*** (19.34)	18.856*** (19.72)
N	4,591	6,029	4,591	6,029	4,591	6,029
R-squared	0.114	0.142	0.061	0.063	0.091	0.112

***, **, * indicate significance at 1, 5, and 10% levels, respectively; *t*-values are in parentheses.

#### 4.6.3 Gender differences

The intergenerational support from adult children may also have gender differences in its impact on the health of older individuals. Statistics indicate that older women have a higher average life expectancy and overall physical health than older men. Therefore, intergenerational support from adult children may exhibit gender differences in its effects on the health and well-being of the older adults. This study divides the sample into two groups: older men and older women, to examine the gender differences in the impact of family support on the health of the older people.

The regression results, as shown in Table 11, indicate that intergenerational economic support from adult children has a significant positive impact on the mental health and positive emotions of older men, while it has a significant negative impact on their negative emotions. In contrast, the effects on the mental health and negative emotions of older women are not significant. This suggests that older men may be more likely to associate economic support with their self-worth; a lack of such support might affect their self-identity and mental health. On the other hand, women may place greater value on family relationships and emotional connections, making them less affected by economic support. The results for the impact of life care and emotional support on both male and female older individuals show similar levels of significance, but the effects of intergenerational support from adult children are more pronounced for men than for women. Therefore, it can be concluded that intergenerational support from adult children has a more significant impact on the health of older men compared to older women.

**Table 11:** Regression results of intergenerational support from adult children for older adults with different gender.

	Mental health		Positive emotion		Depression emotion	
	Male	Female	Male	Female	Male	Female
Financial support	0.082*** (3.01)	0.031 (1.06)	0.026*** (2.91)	0.020** (2.34)	−0.056** (−2.15)	−0.010 (−0.36)
Daily care	−1.728*** (−5.72)	−0.643*** (−2.67)	0.328*** (3.35)	−0.331*** (−4.51)	2.056*** (7.06)	0.973*** (−4.19)
Emotional comfort	0.026** (2.02)	0.024** (1.96)	0.004 (1.00)	0.004 (1.12)	−0.022* (−1.76)	−0.020* (−1.68)
Control	Controlled (Mediate variable is involved)					
Constant	28.356*** (27.33)	24.710*** (23.91)	4.140*** (12.30)	3.758*** (11.95)	15.784*** (15.80)	19.048*** (19.14)
N	5,139	5,481	5,139	5,481	5,139	5,481
R-squared	0.236	0.231	0.079	0.072	0.197	0.199

*, **, *** indicate significance at 1, 5, and 10% levels, respectively; *t*-values are in parentheses.

#### 4.6.4 Personal income difference

The impact of intergenerational support on the health of older adults may also vary depending on the income level of older adults. Older adults with higher income levels, while enjoying support from their children, may be better able to use that support to improve their health, such as choosing better medical care or engaging in more social activities. While older adults with lower income levels may also receive support from their children, they may not be able to take full advantage of this support to improve their health due to economic constraints, and may even face greater stress and health risks.

The regression results are shown in Table 12. First, adult children’s economic support has a significant positive impact on the positive emotions of the older adults with higher income, but has no significant impact on the older adults with lower income. Second, life care has a greater impact on older adults with higher income levels. Although high-income seniors face relatively little financial stress, they may face other types of stress, such as health problems, loneliness, or changing social roles. Child care can provide practical help and emotional support in life, so as to effectively reduce life pressure and improve emotional state. Finally, emotional support has significant positive and negative effects on mental health and negative emotions of the older adults with higher income, but has no significant effect on the older adults with lower income.

**Table 12:** Regression results of intergenerational support from adult children for older adults with different personal income level.

	Mental health		Positive emotion		Depression emotion	
	Below average	Above average	Below average	Above average	Below average	Above average
Financial Support	0.060* (1.84)	0.069*** (2.81)	0.014 (1.40)	0.033*** (4.14)	−0.046 (−1.44)	−0.036 (−1.56)
Daily care	−0.685*** (−2.56)	−1.374*** (−5.24)	0.308*** (3.79)	0.358*** (4.24)	0.993*** (3.76)	1.731*** (7.00)
Emotional comfort	0.018 (1.32)	0.031*** (2.61)	0.006 (1.56)	0.003 (0.73)	−0.011 (−0.86)	−0.028** (−2.51)
Control	Controlled (Mediate variable is involved)					
Constant	26.000*** (22.64)	24.760*** (21.99)	4.151*** (11.91)	3.107*** (8.57)	18.151*** (16.04)	18.347*** (17.25)
N	4,622	5,998	4,622	5,998	4,622	5,998
R-squared	0.186	0.262	0.040	0.087	0.161	0.231

*, **, *** indicate significance at 1, 5, and 10% levels, respectively; *t*-values are in parentheses.

## 5 Discussion

### 5.1 General discussion

This is the first study to integrate three forms of intergenerational support, decomposing mental health into positive and negative emotions, and discussing the impact of intergenerational support on the mental health of older adults based on the main effect and buffering hypotheses. The study revealed that intergenerational support from adult children has a positive impact on the mental health of older adults, which varies according to the form of support received.

Adult children’s financial support has the most robust direct effect on the mental health of the older adults, and its main effect mechanism and buffer mechanism have the same direction of action. Through the enhancement of positive emotions and the weakening of negative emotions, the mental health level is enhanced. Financial support also improves the mental health of older parents by promoting their social participation. The results of this study are an extension of Litwin (13) on the indirect mechanism of economic support on the physical health of the older adults, as well as the work of Li and Wu (61) on the indirect mechanism through which parent–child interaction influences the mental health of older parents. This study contributes to the understanding of the complex relationship involving ‘functional limitations–social interaction–mental health,’ thereby validating the hypothesis that adult children’s financial support improves the mental health of the older parents, with social convoy theory serving as the framework that highlights the critical role of this support in the relationship. This indirect mechanism also represents a novel application of social contagion theory in the realm of social interaction and participation among the older adult.

The effect of adult children’s care support on the mental health of the older adults is not stable (the coefficient of Table 2 and Table 6 is negative, which is different from Table 7). However, after the results are decomposed into the main effect mechanism and the buffer mechanism, the respective effect results of the two mechanisms are stable, that is, adult children’s life care can enhance the positive emotions of the older adults. At the same time, life care also contributed to the negative emotions of the older adults (Tables 3, 4). And Table 4 shows that personal care has a positive promoting effect on the negative emotions of the older adults, while financial support and emotional comfort have a negative impact. Zhao et al. (74) also found similar results in their analysis of the CHARLS data. We speculate that this may be because the older adults who receive care from adult children are mostly disabled older people, who suffer from long-term illness and exhibit relatively high negative emotions. When receiving care, they may feel guilty for dragging down their adult children (74), make the disabled older adults feel ashamed, consequently resulting in negative emotions. As explained by Seeman and Berkman (25) and Lee (75) on intergenerational support, this unbalanced exchange relationship can cause stress or guilt in older adults, with negative effects on mental health. When the inducing effect of negative emotion is higher than the promoting effect of positive emotion, the direction of the influence of adult children’s life care on the mental health of the older adults is negative, and vice versa.

When we extend the connotation of explanatory variable life care to the expected life care of adult children (Table 7), adult children’s care support has a positive impact on the mental health of the older adults. The reason is that the older adults who are expected to receive life care do not actually need adult children’s care, so the expected child “filial piety” main effect exists, while the unbalanced exchange relationship or guilt when receiving adult children’s care has not been felt. Table 11 mainly reflects the main effect of positive emotions. In this way, our results are robust.

The effect of adult children’s emotional comfort support on the mental health of the older adults is very robust. This promotion is not through the main effect, but based on the buffer mechanism (Tables 3, 4), that is, emotional comfort from adult children can alleviate the loneliness of the older adults, and it is not easy to form depression.

This finding provides experimental evidence for the differential effects of intergenerational support on the mental health of older adults, highlighting that each type of support maintains a stable impact within both main effect and buffering models.

### 5.2 Limitations and future directions

This study provides valuable insights into the differential effects of intergenerational support on the mental health of older adults in China. It also expands the application of the main effect model and buffering model by exploring their mechanisms in the context of different forms of intergenerational support. The study conducts a nuanced discussion on how these mechanisms contribute differently to mental health outcomes. However, we recognize certain limitations that warrant consideration.

First, the cross-sectional nature of the data restricts our ability to establish causal relationships between intergenerational support and mental health outcomes. While instrumental variable methods were employed to address potential endogeneity issues, they cannot fully resolve the inherent limitations of cross-sectional data.

Second, although we incorporated comprehensive measures of intergenerational support, more granular data on the frequency, intensity, and quality of support interactions would enable deeper analysis.

Future research would benefit from employing longitudinal or experimental designs to more rigorously examine the causal mechanisms. Additionally, more granular data on the frequency and intensity of support interactions would allow for a more nuanced analysis of these effects. And the different needs of different sub-populations research is a further refinement of the future research direction.

## 6 Conclusion

### 6.1 Main findings

Based on 2018 CHARLS data, this study investigates the mechanisms through which various forms of intergenerational support from adult children affect the mental health of the older adults and explores the heterogeneity of these effects among older individuals with different characteristics and the mediating role of social participation. The main conclusions are as follows:

*Economic Support*: Adult children’s economic support enhances the mental health of older individuals by increasing positive emotions and reducing negative emotions.

*Daily Care*: Daily care from adult children can significantly boost the positive emotions of older individuals, but long-term reliance on such care may induce feelings of guilt and increase negative emotions, ultimately reducing mental health.

*Emotional Support*: Emotional support from adult children improves the mental health of older individuals by alleviating negative emotions.

*Social Participation*: Social participation has a mediating effect on the mental health of older individuals. Economic support from adult children can promote social participation, thereby fulfilling emotional needs at the societal level and enhancing mental health.

Additionally, heterogeneity analysis indicates that intergenerational support from adult children varies among older individuals with different characteristics. Rural older adults have a greater need for emotional support from their adult children. Intergenerational support from adult children has a more pronounced effect on the health of older individuals who are disabled, older adult men, and those from higher-income families.

The main conclusions of this paper elucidate the relationships between intergenerational support from adult children, the mental health of the older adults, and their social participation. These findings provide insights for improving the older adults care system in China, enhancing the role of intergenerational support, and ensuring the mental health of the older people.

### 6.2 Recommendations

*Promote Filial Piety Culture*: Emphasize the role of intergenerational support in the care of older adults. Adult children’s support remains crucial for the well-being of older individuals. Promoting traditional virtues of respecting and supporting the older adults, encouraging adult children to provide economic, care, and emotional resources to their parents, is essential for the quality of older adults’ life. Examples include regular financial support, necessary daily care during illnesses, and frequent communication to alleviate negative emotions.

*Socialize Care Services*: The use of community home care services, including home visits and psychological counseling, can significantly improve the mental health level of the older adult (76) and alleviate their depression (77). Reduce the psychological burden on the older adults by socializing daily care services. Since long-term dependence on adult children for care can lead to feelings of guilt among the older adults, providing primary care services through social systems can effectively reduce these negative emotions. Meanwhile, adult children can offer occasional care to express filial piety, enhancing positive emotions.

*Encourage Social Participation*: Strengthen community functions for older adults care. Communities, as primary venues for daily activities, play a crucial role in helping the older adults obtain external support and improve mental health. Enhancing community facilities to meet the daily and cultural needs of the older adults can significantly boost their mental health. This can include establishing senior activity centers, forming interest groups, and offering volunteer positions to capable older individuals to help them realize personal value.

## Data Availability

Publicly available datasets were analyzed in this study. This data can be found at: https://charls.pku.edu.cn/.
